# Controlling the Evolution of Selective Vancomycin Resistance through Successful Ophthalmic Eye-Drop Preparation of Vancomycin-Loaded Nanoliposomes Using the Active-Loading Method

**DOI:** 10.3390/pharmaceutics15061636

**Published:** 2023-05-31

**Authors:** El Tahra M. Ahmed, Mariam Hassan, Rehab Nabil Shamma, Amna Makky, Doaa H. Hassan

**Affiliations:** 1 Department of Pharmaceutics, College of Pharmaceutical Sciences and Drug Manufacturing, Misr University for Science and Technology, 6th of October City, Giza 12585, Egypt; 2 Department of Microbiology and Immunology, Faculty of Pharmacy Cairo University, Cairo 12613, Egypt; 3 Department of Microbiology and Immunology, Faculty of Pharmacy, Galala University, New Galala City, Suez 43511, Egypt; 4 Department of Pharmaceutics and Industrial Pharmacy, Faculty of Pharmacy Cairo University, Cairo 12613, Egypt

**Keywords:** active-loading method, liposome dialysis cassette, vancomycin, antimicrobial resistance

## Abstract

Vancomycin is the front-line defense and drug of choice for the most serious and life-threatening methicillin-resistant Staphylococcus aureus (MRSA) infections. However, poor vancomycin therapeutic practice limits its use, and there is a consequent rise of the threat of vancomycin resistance by complete loss of its antibacterial activity. Nanovesicles as a drug-delivery platform, with their featured capabilities of targeted delivery and cell penetration, are a promising strategy to resolve the shortcomings of vancomycin therapy. However, vancomycin’s physicochemical properties challenge its effective loading. In this study, we used the ammonium sulfate gradient method to enhance vancomycin loading into liposomes. Depending on the pH difference between the extraliposomal vancomycin–Tris buffer solution (pH 9) and the intraliposomal ammonium sulfate solution (pH 5–6), vancomycin was actively and successfully loaded into liposomes (up to 65% entrapment efficiency), while the liposomal size was maintained at 155 nm. Vancomycin-loaded nanoliposomes effectively enhanced the bactericidal effect of vancomycin; the minimum inhibitory concentration (MIC) value for MRSA decreased 4.6-fold. Furthermore, they effectively inhibited and killed heteroresistant vancomycin-intermediate S.aureous (h-VISA) with an MIC of 0.338 μg mL^−1^. Moreover, MRSA could not develop resistance against vancomycin that was loaded into and delivered by liposomes. Vancomycin-loaded nanoliposomes could be a feasible solution for enhancing vancomycin’s therapeutic use and controlling the emerging vancomycin resistance.

## 1. Introduction

According to the WHO, there are 2.2 billion of the visually affected population across the world [1,2]. Global alarm for subsequent dramatic socioeconomic disasters has urged the need for deep-insight research of causes and possibly reliable ways of control and prevention. Infective eye conditions, which are common and could be hazardous sight conditions, are caused by a wide spectrum of microbes; however, bacterial keratitis is the most reported ophthalmic infective microorganism, with a high incidence of gram-positive bacteria in particular [3,4,5,6]. Staphylococcus aureus is the major causative microorganism of serious ophthalmic infection, and its severity is not only due to its virulence factors but also due to its capability to develop multidrug resistance [3,7].

Vancomycin is the golden-standard therapy for treatment and management of bacterial keratitis caused by gram-positive and multi-infective keratitis [7,8,9,10,11].

Nevertheless, vancomycin’s clinical use has significant limitations related to its poor tissue-penetration profile, its pharmacokinetic properties, its toxicity, and the recent emergence of selective microbial resistance. Moreover, eye barriers for drug delivery: tear film, corneal-epithelium-cell tight junction, and molecular weight cut-off limitations, further weaken vancomycin’s therapeutic efficacy and promote the development of selective vancomycin resistance.

Several approaches have been employed to enhance the therapeutic-toxicity index of vancomycin by modifying its physicochemical properties and, hence, its pharmacokinetic profile. Among them are nano-based drug-delivery systems.

Nanovesicles as drug-delivery platforms have a proven capability to enhance on-site drug availability, hence therapeutic efficacy, and reduce potential drug toxicity [12,13,14].

Vancomycin has been loaded into various nanoparticles, including lipid, metallic, and polymeric nanoparticles. However, vancomycin’s characteristic physicochemical properties, a high molecular weight (1449.3 Da), a hydrophilic nature, and structural complexity with fulfillment of many reactive H-donating/accepting groups, impede its efficient loading into liposomes [15,16,17,18,19,20,21,22,23,24,25,26].

Among the wide varieties of nanovesicles, liposomes, which are cell-like structured nanovesicles, have gained a special, unique interest as drug carriers due to their high biocompatibility, safety, ability to load a wide range of drugs with different physicochemical parameters, and ability to be easily functionalized for specific drug targeting. And so, liposomes were crowned the most FDA-approved nanocarriers for drug delivery [27,28,29,30].

Additionally, liposomes specifically enhance ocular drug bioavailability. Their lipidic nature allows corneal penetration and drug delivery to various eye tissues and also prolongs contact time at the eye surface. Liposomes are safe, nonirritating, and nontoxic for the eye as well [31].

Vancomycin-loaded liposomes have markedly enhanced vancomycin’s biodistribution, pharmacokinetics, and pharmacodynamic profile. Additionally, their intracellular delivery ability boosted the vancomycin bactericidal effect on MRSA strains and significantly decreased the highest MIC threshold [24,32,33,34,35,36].

Nevertheless, the low encapsulation efficiency of liposomes for vancomycin (9–20%) using the passive loading technique has overshadowed its golden delivery advantages [24,35,36].

The active drug-loading method is an advanced technique that was proposed as a controllable technique to enhance the liposomal drug-loading capacity through concentrating the liposomal payload.

In principle, the active/remote-loading technique is based on preparing drug-free liposomes at a certain ionic concentration/pH. Exchanging a liposome’s surrounding solution with a drug solution of a different ionic concentration/pH would initiate an ionic imbalance in the liposomal system. This ionic stress enforces ionic movement across the liposomal membrane, with a molecular exchange between intraliposomal and extraliposomal molecules. This allows active drug passage from outside to inside liposomes [37,38,39,40,41,42,43,44].

Vancomycin can adapt to different ionization states according to pH change [45,46,47].

**Figure 1:**
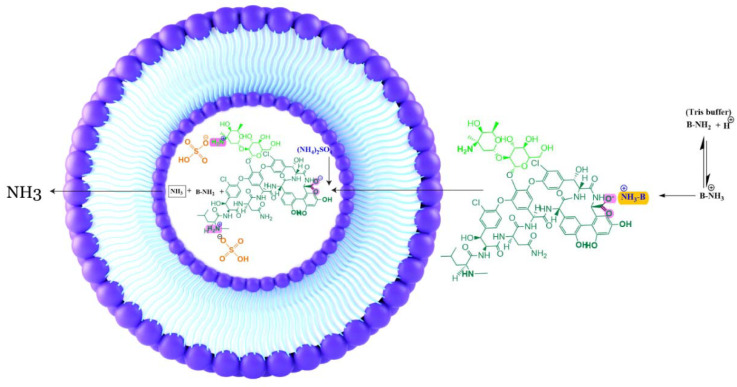
Active loading of vancomycin using ammonium sulfate gradient at pH of 9, while vancomycin carboxylic group, the only ionized group, was readily neutralized and formed carboxylate salt with the acidic form of Tris, Tris-HCl. Upon vancomycin passage to the insides of liposomes at pH of 4–5, vancomycin transformed to bisulfate salt while neutral ammonium molecules passed through the liposomal membranes to the outsides.

Changing the degree of ionization subsequently alters the degree of vancomycin hydrophilicity and its polar/nonpolar surface area. This suggests possible good control of vancomycin entrapment within liposomes, depending on the difference in pH between the insides and outsides of liposomes. Vancomycin transformation from the neutral, zero-charge state on the outsides of liposomes to a higher ionization state within liposomes could reasonably limit its lipid permeability, with consequent good accumulation inside the liposomes (Figure 1).

In our study, vancomycin-loaded liposomes were prepared according to the active-loading process. The prepared liposomes were evaluated for vancomycin entrapment efficiency, particle size, and zeta potential, and they were optimized according to a statistical design. The optimized vancomycin-loaded liposomes were morphologically examined using a transmission electron microscope. The physical state of vancomycin in the prepared liposomes was examined using differential scanning calorimetry. The activity of vancomycin-loaded liposomes on vancomycin antimicrobial activity against bacterial populations with variable resistance mechanisms, multiple-drug resistance, and biofilm formation in comparison to vancomycin solution and blank liposomes was also examined.

## 2. Materials

Vancomycin HCl was kindly gifted by the Egyptian national organization for drug control and research. Soybean phosphatidylcholine, cholesterol, Trizma, Tris HCl, and Tween^®^20 were used (Sigma, Roedermark, Germany). Ethanol of HPLC grade, chloroform of 99.8% analytical-reagent grade, and acetic acid were also used (ThermoFisher, Waltham, MA, USA), as were ammonium sulfate (Honeywell, Offenbach, Germany), Sephadex G50 fine (GE Healthcare, Amersham, UK) NaCl, and NaOH (El-Nasr pharma, Sohag, Egypt).

## 3. Methodology

### 3.1. Cassette Construction

**Figure 2:**
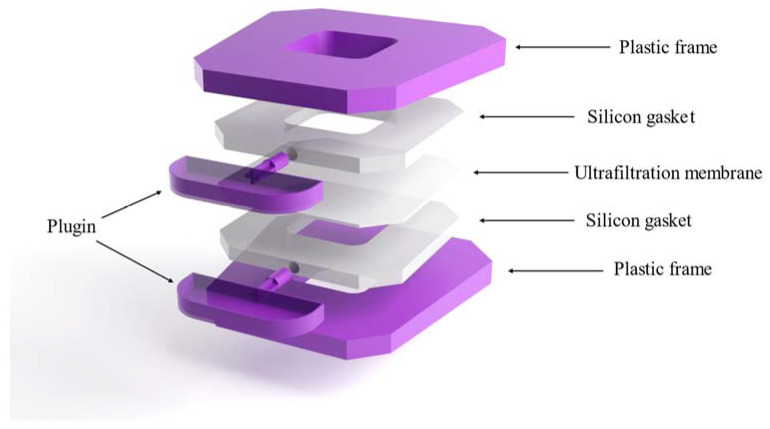
Diagrammatic representation of the modified liposome dialysis cassette.

The liposome dialyzer cassette was prepared as detailed by Adamala and coworkers, with some modifications, considering all detailed construction notes and troubleshooting in their protocol [48]. In our work, we used a Slide-A-Lyzer G2 Dialysis Cassette (Thermo Scientific, Swindon, UK), since its construction is simpler and more convenient for laboratory use with our modifications in comparison to the classic dialysis cassette in the Adamala cassette model. A representative diagram of our cassette is illustrated in Figure 2, and detailed construction steps are included in the Supplementary Data (Figures S1–S4). Additionally, cassette cleaning and storage details are included, considering that cleaning the ultrafiltration membrane is critical to avoid pores clogging with lipids.

### 3.2. Liposome Preparation

**Figure 3:**
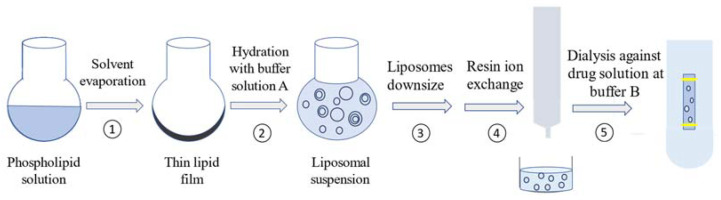
Schematic representation of preparation steps of vancomycin-loaded liposomes.

A schematic representation of vancomycin active loading steps was illustrated in Figure 3. 

#### 3.2.1. Preparation of Drug-Free Liposomes

Liposomes were prepared using the thin-lipid-film hydration method [44,49]. Accurate volumes of cholesterol and soy-lecithin stock solutions were mixed in a 500 mL round flask, and a thin lipid film was obtained after chloroform evaporation using a rotary evaporator (Heidolph, Schwabach, Germany) under vacuum at 100 rpm and 35 °C to avoid lipid degradation for almost 2 h. The liposomal suspension (5 mM) was obtained upon hydration of the thin lipid film with 20 mL of ammonium sulfate solution (250 mM) (Honeywell, Offenbach, Germany) preheated to 65 °C. The liposomes were transferred to glass test tubes and tumbled overnight using a tumbler mixer (LabDex, Farnborough, UK) at 20 rpm to obtain giant unilamellar vesicles [48]. Preparation details of the lipid stock solutions and the Tris buffer solution are illustrated in the Supplementary Data.

#### 3.2.2. Liposome Downsizing

Liposomes were downsized with VCX 750 Ultrasonic Processors (Sonics VCX 750, Newtown, CT, USA) using 3 mm microtips. The sonication process was operated at a 35% sonication amplitude and a pulse mode of 50% of the duty cycle (one-minute interval) for six successive sonication cycles, with a total sonication time of 6 min at 25 °C. The depth of the sonicator tip in the liposomal suspension, the volume of the sample (10 mL), and the dimensions of the liposomal container (15 mL falcon tube) were fixed parameters throughout this experiment. Sonication was carried out in an ice bath to avoid lipid degradation during the sonication process. After sonication, the liposomal suspension was subjected to centrifugation at 5000 rpm for 30 s to eliminate possibly shaded metals from the sonicator tip.

#### 3.2.3. Gel Filtration and Ion Exchange

Removal of extraliposomal ammonium ions to initiate ammonium-ion and pH gradients was carried out using an ion-exchange resin. Sephadex G50 fine, as an exchange medium, was loaded into PD-10 parallel columns (Cytiva, Marlborough, MA, USA), and the liposomal suspension (2.5 mL) was applied gently onto a gel bed, where NaCl solution (350 mM) was used as an eluting solution. Eluted samples were collected as various fractions (each 1 mL) and subjected to analysis to determine the lipid concentrations and ascertain the removal of extraliposomal ammonium ions. Based on spectrophotometric analysis (UV-1800 Spectrophotometer; Shimadzu, Germany), the phosphorus determination method was used to determine the total lipid concentration [50]; meanwhile, excess ammonium ions were determined using the Berthelot reaction [51].

#### 3.2.4. Drug Loading

Vancomycin solution with various concentrations was prepared by dissolving vancomycin HCl powder in 200 mM of Tris buffer at a pH of 9, and 3 mL of the prepared solution was loaded into the drug-cassette chamber using a pipette, where 3 mL of the previously prepared liposomes was loaded into the liposome chamber using a glass pipette. The cassette was tumbled at 80 rpm using a vertical rotating mixer (LabDex, London, UK) at room temperature for 24 h, and the drug-chamber content was replaced (1, 2, or 3 times). After loading, the dialyzer contents were removed, and the drug content was determined indirectly with spectrophotometric analysis at λ_max_ 280 nm [52].

### 3.3. Evaluation of Vancomycin-Loaded Liposomes

#### 3.3.1. Particle Size and Zeta-Potential Determination

Particle size, size distribution, and surface charge were analyzed depending on hydrodynamic radius measurements of dynamic light scattering (DLS). The isotropic morphology assumption of liposomes makes DLS a reliable method for size, dispersion, and zeta-potential analysis for liposomes. A Malvern Instruments Zetasizer Nano Series (Malvern, UK) was used for DLS analysis; the refractive index was set as 1.335 and the medium viscosity at 1.020 cP. The prepared vancomycin-loaded liposomes were subjected to dilution (1 to 10 mL) with bidistilled water, and measurements were carried out at room temperature. Results were represented as the intensity means of triplicate measurements of the sizes, dispersity, and zeta potential of vesicles.

#### 3.3.2. Entrapment Efficiency

Vancomycin-loaded liposomes were centrifuged at 20,000 rpm at −4 °C for 2 h using a cooling centrifuge (Sigma 3-18KS, Roedermark, Germany), and the supernatant was separated to quantify the free vancomycin. The total amount of vancomycin was quantified by dissolving 0.5 mL of the prepared vancomycin-loaded liposomes in 2 mL of precooled ethanol at −20 °C [53]. Entrapment efficiency was calculated using the following equation:

$$\mathrm{EE}\%=\frac{Total\;amount\;of\;drug-Amount\;of\;free\;drug}{Total\;amount\;of\;drug}\times 100$$

Spectrophotometric analysis was used to measure the vancomycin concentration at λ_max_ = 280 nm, based on the calibration curve that was previously constructed at the vancomycin concentration range of 15–200 µg/mL.

#### 3.3.3. Morphological Characterization

Transmission electron microscopy (TEM) imaging was used for direct identification of the shapes, surface properties, and sizes of the selected vancomycin-loaded liposomes. In brief, a drop of the liposomes was placed onto the TEM mesh grid and covered with carbon supporting film, allowing the liposomal suspension to be adsorbed onto the grid but not dried; then, the sample was blotted off, and uranyl acetate stain (2% solution) was applied to the grid. Excess stain was drained out, and the grid was left for complete drying at room temperature to become ready for imaging.

#### 3.3.4. Differential Scanning Colorimetry (DSC)

The selected vancomycin-loaded liposomes, blank liposomes, and vancomycin HCl powder were subjected to thermal examination using DSC-50 (Shimadzu, Duisburg, Germany) to determine enthalpy changes associated with phase transitions due to the entrapment process. An accurately weighed amount of each sample was placed in an aluminum cell, and its thermogram was recorded. DSC was carried out at a heating rate of 10 °C/min from 25 °C to 300 °C under a nitrogen flow of 25 mL/min.

### 3.4. Experimental Design and Statistical Analysis

To assess the magnitude and significance of the main effect of each predictor variable and the possible interactions a screening experimental model (fractional factorial design) was applied using JMP Pro (SAS Institute, Cary, NC, USA). According to our study, the cholesterol percentage (10%, 30%), the drug:lipid (D/L) molar ratio (0.05, 0.2), and the number of drug-solution exchanges (1, 2, 3) were the predictor variables.

The linear-regression-analysis method was selected to model the relationship between the predictor variables and the measured responses for entrapment efficiency (EE%), particle size, and zeta potential.

### 3.5. Antimicrobial Activity

#### 3.5.1. Antibacterial-Activity MIC and MBC

The antibacterial activities of vancomycin solution, vancomycin-loaded liposomes, and blank liposomes were determined against the following bacterial strains: methicillin-resistant *Staphylococcus aureus* (MRSA USA300), vancomycin-intermediate-resistant *Staphylococcus aureus* (hVISA OP933674), *Staphylococcus epidermidis* ATCC 35984, and *Staphylococcus epidermidis* ATCC 12228 [54]. The broth-microdilution method was used according to the guidelines of the Clinical and Laboratory Standards Institute to determine the minimum inhibitory concentration (MIC) and the minimum bactericidal concentration (MBC) [55,56].

#### 3.5.2. Effect of the Optimized Vancomycin-Loaded Liposomes on the Antibiofilm Activity of Vancomycin

The antibiofilm activity of the vancomycin-loaded liposomes was compared to that of the free vancomycin solution. The vancomycin solution and the vancomycin liposomes were tested at concentrations below the determined MIC. Biofilm-forming bacterial strains (methicillin-resistant Staphylococcus aureus (MRSA USA300) and Staphylococcus epidermidis ATCC 35984) were used in this assay [57]. 

##### Biofilm Inhibition Assay

The biofilm inhibition assay was performed as previously described in the literature [58]. Briefly, the bacterial suspension (10^8^ CFU/mL) in tryptic soy broth (TSB) was loaded in a flat-bottom 96-well plate (200 μL/well). Different concentrations of the tested solution/liposomes were added to the loaded wells (20 μL/well). Nothing was added to the control wells (untreated wells, 100% reference values). The plates were then incubated at 37 °C for 24 h. After incubation, the optical density (OD_600_) of the grown cultures was measured with a spectrophotometric plate reader (Biotek, Synergy 2, Winooski, VT, USA). The formed biofilms were quantified using the crystal violet assay as follows: wells were washed three times with saline and then dried. The dried biofilm was then stained with crystal violet (0.1% *w/v*, 200 μL/well) for 30 min at room temperature. The wells were then washed three times with distilled water and dried. The crystal violet in the stained biofilm was solubilized by adding absolute ethanol (200 μL/well) and incubating for 20 min at 4 °C. The OD_550_ of the crystal violet solutions was measured and divided by the OD_600_ of the grown cultures for normalization. This experiment was repeated three independent times. The biofilm inhibition percentage was calculated using the following equation [57]:

$$Biofilm\;inhibition\;\%=\frac{OD\;Control-OD\;Test}{OD\;Control}\times 100$$

##### Biofilm Eradication Assay

The ability of the tested solution/liposomes to eradicate the previously established biofilm was investigated. This experiment was performed as previously described in the literature [58]. A bacterial suspension in TSB (10^6^ CFU/mL) was loaded in a flat-bottom 96-well plate (200 μL/well) and incubated at 37 °C for 24 h. After incubation, the OD_600_ of the grown cultures was measured. The wells were carefully emptied using aspiration. Different concentrations of the tested solution/liposomes were prepared in fresh TSB and were added to the biofilm plate (200 μL/well). Nothing was added to the biofilm control wells (untreated biofilm, 100% reference value). The plate was then reincubated at 37 °C for 24 h. After the treatment period, crystal violet staining and measurement were performed as described above. This experiment was repeated three independent times. The biofilm eradication percentage was calculated using the following equation [57]:

(1) $$Biofilm\;eradication\;\%=\frac{OD\;Control-OD\;Test}{OD\;Control}\times 100$$

#### 3.5.3. In Vitro Resistance Study

Methicillin-resistant *Staphylococcus aureus* (MRSA USA300) and *Staphylococcus epidermidis* ATCC 35984 were used in this experiment. The serial-passages resistance experiment was performed as previously described in the literature [59,60]. Briefly, bacterial strains were cultured with serial concentrations of either vancomycin-loaded liposomes or vancomycin solution in 96-well plates. The bacterial cultures were passaged daily for 25 days, and the broth microdilution method was used to determine the MIC and MBC of each passage. In each passage, the serial concentrations of the vancomycin-loaded liposomes/solution were inoculated using inoculum from the wells, corresponding to the 0.5 MIC determined from the previous passage. The fold change in the MIC/MBC was calculated in each passage and compared to the initially determined MIC/MBC. Resistance was defined as a greater than or equal to a 4-fold increase in the MIC [59,60]. This experiment was repeated three independent times.

### 3.6. In Vitro Eye-Safety Test

The hen’s egg test, chorioallantoic membrane (HET-CAM), was used as an in vitro examination of the potential irritation effect of optimized vancomycin-loaded liposomes on the surface of the eyes [61,62,63].

To simulate their impact on the ocular mucosal membrane when used in vivo, the selected vancomycin-loaded liposomes were smeared directly onto the egg CAM, and the membrane was watched for any modification or change. This test had three steps [64,65], which were as follows.

#### 3.6.1. Preparing the Eggs

At 37.5 °C and 66% relative humidity, freshly fertilized chicken eggs were incubated for 10 days. In order to prevent the embryo from adhering to one side, the eggs were rotated three times daily throughout this time. The eggs were candled after 10 days to check the viability of the embryos and to mark the air gaps with a pencil. Eggs that were not viable were discarded. With a dental rotary saw and scissors, the eggshell covering the air space was taken off before use.

The inner membrane was exposed, and saline was sprayed on it to moisturize it. The inner membrane was pulled away with forceps just before usage to reveal the CAM, which was then deemed prepared for the application of the test ingredients.

#### 3.6.2. Application of Test Materials

The vancomycin-loaded liposomes, the vancomycin aqueous solution, and the blank liposomes were the three systems that were tested. A 10% NaOH solution was utilized as a positive control, whereas saline was employed as a negative control. The exposed CAM received a precise 0.3 mL of each tested system, with at least 50% of its surface covered for 5 min.

#### 3.6.3. Visual Analysis

The CAM was visually examined for any vascular changes, such as hemorrhage, hyperemia, lysis, extravascular coagulation, or intravascular thrombosis, following the application of the test chemicals. Any of these changes would indicate a potential propensity for the tested compounds to harm the eye’s mucosal membrane when used in vivo.

## 4. Results and Discussion

Vancomycin-loaded nanoliposomes were successfully prepared using the active-loading method. The effects of various formulation variables were studied using JMP Pro software. The data set was checked for meeting linear regression assumptions using residual-prediction plots of the results for EE%, particle size, and zeta potential. Based on the distribution analysis, errors were randomly scattered around the mean of zero and normally distributed (normality was checked using the Shapiro–Wilk (W) and Anderson–Darling (A2) statistical tests at *p*-values < 0.05), confirming constant variance with the independence of observations to one another. Additionally, data were checked for outliers using quantile distribution (tail quantile 0.25 and Q 1.5), and there were no outliers detected. By employing regression analysis, the standard least squares model fit the data with R^2^ = 0.992654, R^2^ adjusted = 0.973065, and a Root Mean Square Error (RMSE) = 1.424234.

### 4.1. Entrapment Efficiency

Through 10 different vancomycin-loaded nanoliposomes, the liposomes showed successful vancomycin entrapment, with the EE% ranging from 32.1% to 65% (Table 1).

Encapsulation of vancomycin at high efficiency (up to 65%) reflected successful vancomycin diffusion across the lipid membranes of the preformed liposomes, with subsequent good accumulation within the cores. Despite vancomycin’s hydrophilicity, as a tricyclic heptapeptide and a member of the macrocyclic drugs, its own capability to pass through phospholipid bilayers is related to its capability to make conformational changes according to solvent structural preference rather than its overall low lipophilicity [66,67,68,69].

Vancomycin could equilibrate between two structural forms, monomeric and dimeric, of different geometric orientations and polar/nonpolar surface area, hence its lipophilicity [70].

**Figure 4:**
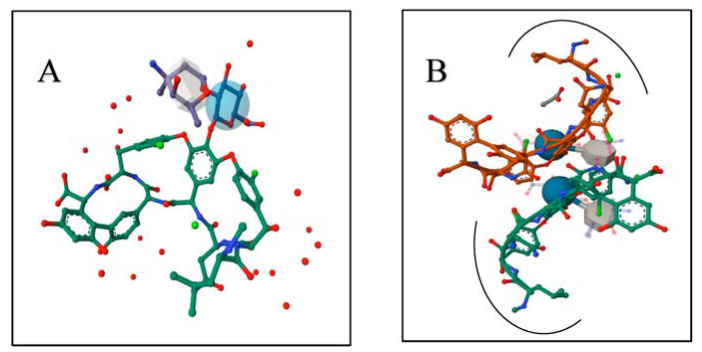
(**A**) Crystal structure of vancomycin monomer [76]. (**B**) Crystal structure of back-to-back vancomycin dimerization, where two vancomycin monomers are connected at the peptide backbone. The arched line points out the inward-oriented vancomycin hydrophobic wall that encompasses the aromatic ring of phenyl glycine, the hydroxy tyrosine residue, and the isobutyl group of leucine residue [74].

Back-to-back vancomycin dimer, the readily formed dimerization form of vancomycin, dose shielded the vancomycin’s polar peptide backbone by forming four amide–amide H-bonds along the peptide backbone of two monomers (Figure 4). Vancomycin back-to-back dimerization effectively shielded 15% of each vancomycin monomer surface area, whereas about 52% of this buried area was polar [70,71,72,73,74]. Furthermore, the geometric orientation of the vancomycin hydrophobic wall, the aromatic ring of phenyl glycine, the hydroxy tyrosine residue, and the isobutyl group of the leucine residue of the vancomycin dimer further protected the vancomycin molecule from water molecules [75].

Therefore, the vancomycin dimeric form was supposed to be a superior lipophilic form of vancomycin that allowed vancomycin lipid-membrane permeability.

Moreover, at the selected pH (pH = 9), vancomycin was present at its least polarized state and polar/nonpolar surface area, in which the only ionized group, the terminal carboxylic group, was readily neutralized by the Tris-HCl counterion to form ammonium carboxylate salt [77].

The subsequent accumulation of vancomycin within liposomes was a result of the transformation of vancomycin to a higher-ionization and less-lipid-permeable form. By proceeding of vancomycin molecules to the inside of liposomes, at a pH of 5–6 of ammonium sulfate, the primary amine of the vancosamin sugar and the secondary amine of leucine residue were protonated, with subsequent formation of sulfate salt of the positively charged divalent vancomycin molecule.

**Figure 5:**
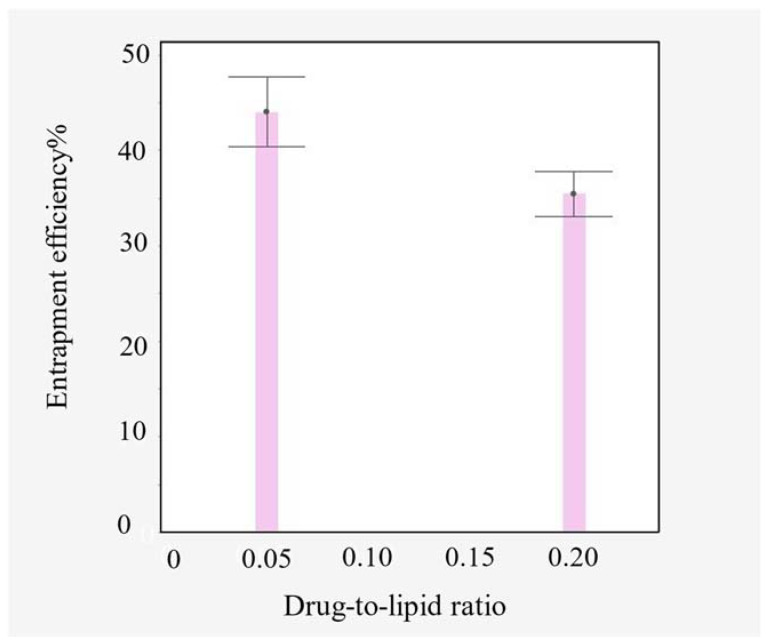
Entrapment efficiency as a function of the drug-to-lipid ratio; error bar was constructed using one standard error.

By running the experimental model analysis, it was found that the EE% of vancomycin is inversely affected by the molar D/L ratio (*p*-value = 0.00166), with consistently higher EE% values at low D/L ratios (Figure 5).

**Figure 6:**
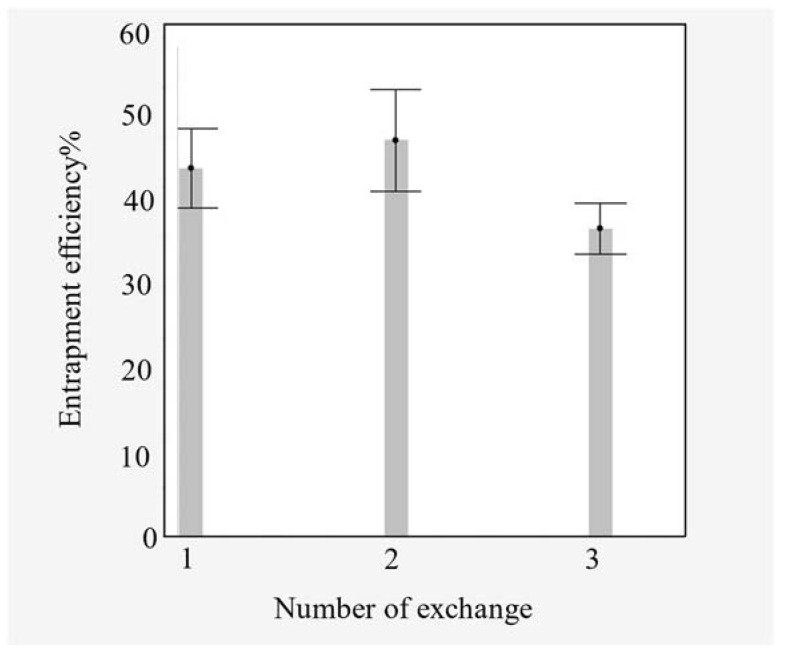
Entrapment efficiency as function number of drug-solution exchanges; error bar was constructed using one standard error.

Additionally, the number of exchanges of vancomycin solution in the drug chamber significantly affected the EE% (*p*-value = 0.00156) (Figure 6). Although there was no significant difference in the EE% between one or two exchanges of vancomycin solution, three exchanges negatively affected the EE%.

Despite cholesterol content (10–30%) having no chief effect on vancomycin entrapment, the predictor variables’ interaction profile revealed a significant interaction between the cholesterol percentage and the D/L ratio at a low level (0.05) (*p*-value = 0.02619).

Contour prediction plots were employed to investigate this interaction and to optimize the vancomycin loading process. The contour plot of the EE% was displayed as a function of the cholesterol percentage and the D/L ratio, and the concentric contour lines connected different combinations of cholesterol percentages and drug-to-lipid ratios with the same entrapment values.

**Figure 7:**
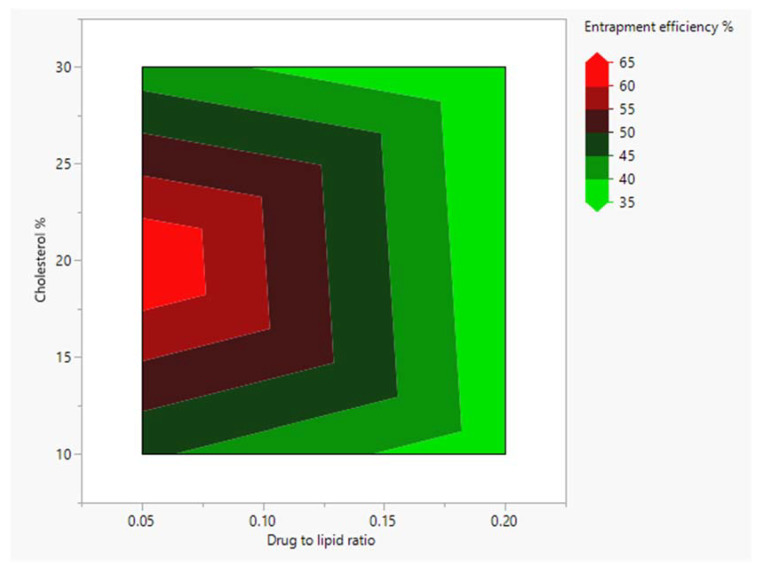
Contour plot of entrapment efficiency as a function of cholesterol percentage and drug-to-lipid ratio. The colored contour bands represent different ranges of entrapment value.

The contour plot illustrated a great variability in vancomycin’s EE% at a 0.05 D/L ratio with changes in cholesterol percentage. Meanwhile, this variability in EE% was diminished at high levels of D/L ratios with overall low entrapment efficiency. Additionally, this showed that a cholesterol content of 20% would give the highest entrapment efficiency at low D/L ratios (Figure 7).

Additionally, the predictor variables’ interaction profile revealed a significant interaction between the D/L ratio and the number of exchanges of vancomycin solution (*p*-value = 0.01055). Hence, the optimum number of drug-solution exchanges depends on the level of the D/L ratio.

**Figure 8:**
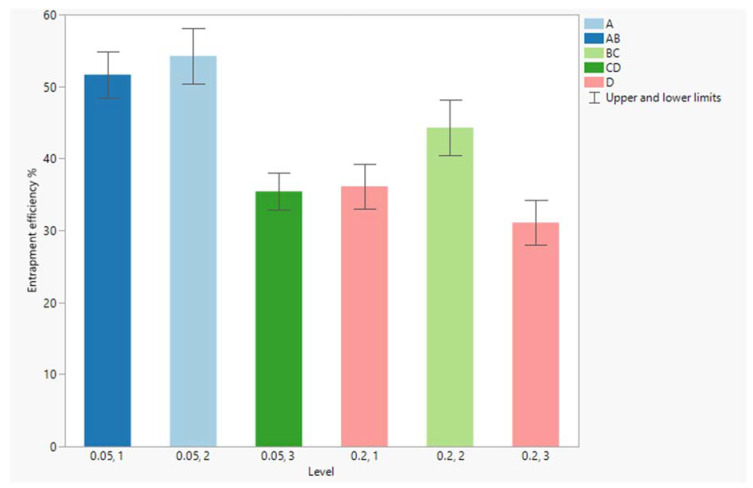
Vancomycin entrapment efficiency at different combinations of drug-to-lipid ratio and numbers of drug-solution exchanges; combinations that have the same connecting letter are not significantly different.

To explore this interaction, we further studied the effects of different combinations of numbers of exchanges at each level of the D/L ratio on vancomycin entrapment efficiency, using pairwise comparisons (Tukey HSD test) (Figure 8).

For the low D/L ratio (0.05), the results revealed no significant difference in vancomycin entrapment between single and double exchanges with the vancomycin solution in the drug chamber, while triple exchanges resulted in a marked decrease in vancomycin entrapment. Meanwhile, for the high D/L ratio (0.2), the maximum vancomycin entrapment was obtained with two subsequent vancomycin-solution exchanges, while single and triple exchanges significantly achieved lower entrapment in comparison to the double exchanges (*p*-values of 0.0487 and 0.0126, respectively).

This achievement of high vancomycin entrapment at a low D/L ratio from a single exchange of its solution in the drug chamber, in contrast to that of the high D/L ratio, has a special concern, since these data point out an additional vancomycin diffusion barrier at its higher concentrations (through our experiments, a high D/L ratio was achieved by increasing the vancomycin concentration in relation to the constant lipid concentration). At high vancomycin concentrations, vancomycin forms self-supracomplexes with molecular weights of up to 12,000 KDa [70,78]. Dissociation of these compounds to the dimeric form becomes an additional diffusion-limiting factor for vancomycin loading.

### 4.2. Particle Size

#### Hydrodynamic Sizes and Zeta Potential of Liposomes as Measured with Dynamic Light Scattering (DLS)

Dynamic light scattering, or photon correlation spectroscopy, was used for the determination of hydrodynamic sizes and surface charges across our different liposomes (Table 1). Different vancomycin-loaded nanoliposomes showed average sizes ranging from 141 to 353 nm, while the optimized vancomycin-loaded nanoliposomes had an average size of 155 nm. In addition, the liposomes had negative zeta potential, ranging from −18.5 to −41 mV, and the optimum vancomycin-loaded nanoliposomes had a zeta potential of −34 mV. The high value of negative zeta potential indicates good dispersible stability of the formulated liposomes as a result of charge repulsion among dispersed particles.

**Figure 9:**
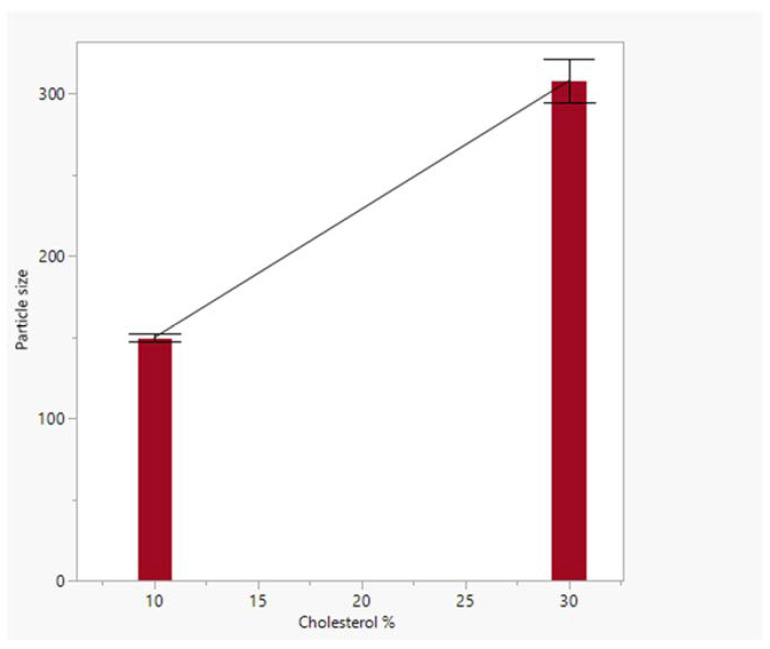
Particle sizes at different levels of cholesterol; error bar was constructed using one standard error.

Statistical analysis of liposome size as a response to variability in cholesterol content, the D/L ratio, and the number of drug-solution exchanges showed that cholesterol content significantly affects liposome size (*p*-value of 0.004) (Figure 9). At the same time, the D/L ratio and the number of drug-solution exchanges had no significant impact on liposome size.

Unlike entrapment efficiency, cholesterol percentage predominantly affects liposome size. This is related to what is defined as the condensing effect of cholesterol, where cholesterol minimizes the tilting angle of the lipid acyl chain in the liposomal membrane, resulting in an increase in membrane thickness. The condensing effect of cholesterol is intimately related to its molar percent in the lipid membrane, which is reflected clearly in the liposome’s size [79,80,81,82].

### 4.3. Zeta Potential

The prepared vancomycin-loaded nanoliposomes, with their variability, expressed a high negative value of zeta potential, ranging from −18.5 to −41 mV, owing to the negatively charged phosphate group of soy lecithin [83,84].

**Figure 10:**
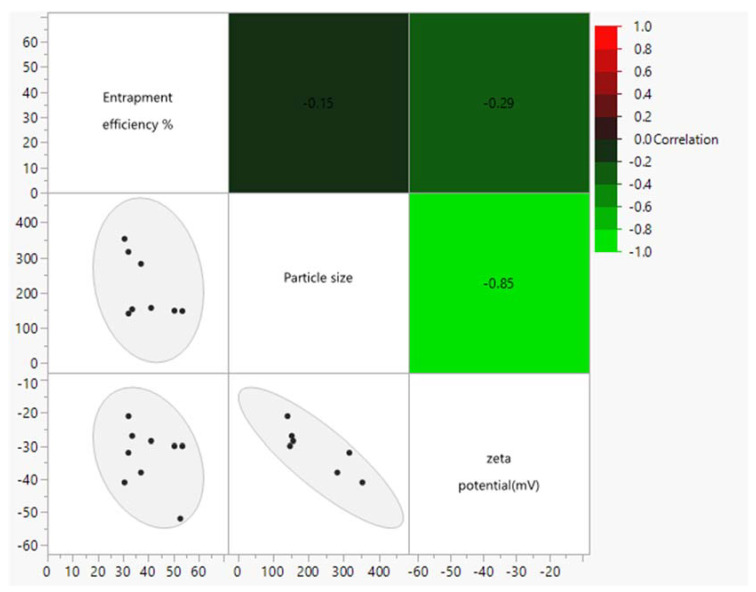
Multivariate scatterplot matrix of entrapment efficiency, particle size, and zeta potential illustrates that there is a strong negative correlation between particle size and zeta potential (R = −0.85) and there is a correlation between neither and entrapment efficiency.

Statistical analysis of the effects of variability in cholesterol content, the D/L ratio, and the number of drug-solution exchanges on zeta potential confirmed no significant effect for any of these factors. However, multivariate analysis of entrapment efficiency, particle size, and zeta potential illustrated a strong inverse correlation between zeta potential and particle size (R^2^ = −0.85, *p* = 0.0068) (Figure 10).

Based on the aforementioned results for different vancomycin-loaded nanoliposomes’ EE% values, particle sizes, and zeta potential, the selection of the optimized vancomycin-loaded nanoliposome, principally, depended on the EE% value, as the particle sizes and zeta potential of different liposomes fell within the accepted range for our applications. Therefore, according to the JMP-prediction-profile liposomal formula of 20% cholesterol, a 0.05 D/L ratio, and a number of vancomycin-solution exchanges of two, the optimum vancomycin-loaded nanoliposome is that with a vancomycin EE% of 65%.

### 4.4. Physicochemical Characterization of Optimized Vancomycin-Loaded Nanoliposomes

#### 4.4.1. Liposome Size and Morphology Measurements with Transmission Electron Microscopy (TEM) Imaging

**Figure 11:**
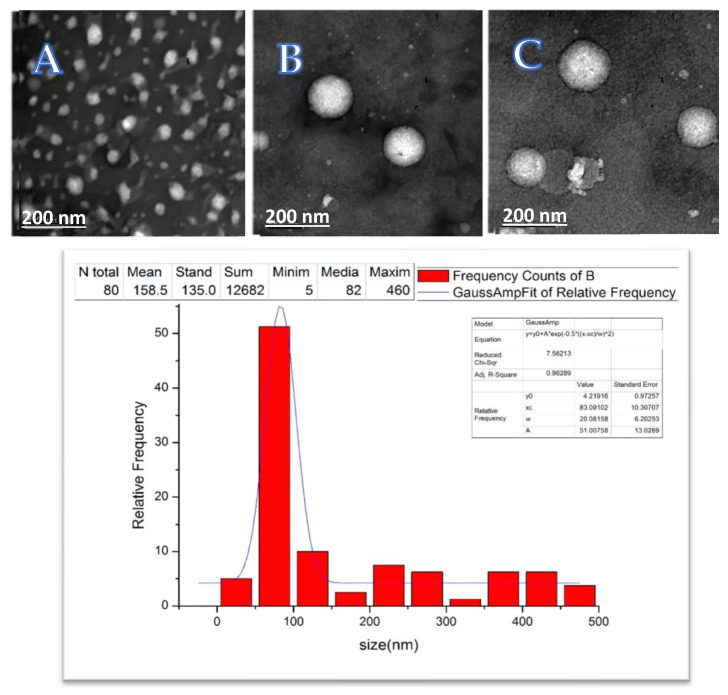
Top panel: transmission electron microscopy (TEM) images (**A**) illustrating homogenous unaggregated vesicles and consistent spherical morphology of the vancomycin-loaded nanoliposomes (**B**,**C**). Bottom panel: histogram analysis of the particle sizes, showing mean liposome size (median) of 82 nm as measured with TEM.

TEM imaging revealed the consistent spherical morphology of the vancomycin-loaded nanoliposomes. Most of the measured liposomes had diameters ≤ 200 nm, with a dominant diameter (median) of 82 nm with no aggregates (Figure 11).

#### 4.4.2. Physicochemical Thermodynamic Stability Determination Using Differential Scanning Calorimetry (DSC)

**Figure 12:**
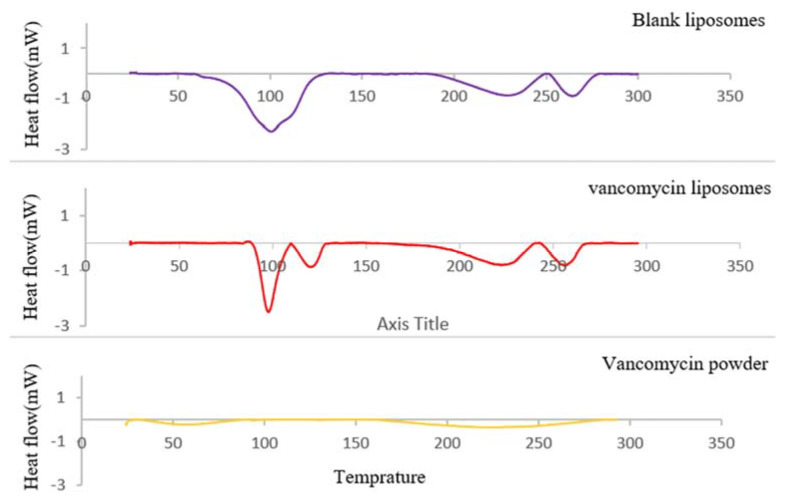
Differential scanning calorimetry (DSC) of vancomycin, vancomycin-loaded liposomes, and blank liposomes.

Scanning the thermodynamic stability of vancomycin in its crystalline-powder and liposome-loaded forms illustrated significantly different thermal behaviors of both (Figure 12). Vancomycin powder undergoes two major enthalpy changes upon a heating scan; the first endothermic peak (onset at 39.9 °C) is related to the thermal degradation of dehydration and the oxidative demethylation of vancomycin molecules [53], while the second endothermic broad peak (onset at 169.5 °C) refers to the melting-point transition of the vancomycin amorphous structure [85].

The DSC thermogram of the vancomycin-loaded nanoliposomes showed different thermal behaviors of vancomycin and the disappearance of its two characteristic peaks. Furthermore, the similarity between the thermotropic behavior of blank liposomes and that of vancomycin-loaded nanoliposomes allows inference of high vancomycin encapsulation in a molecular state within the liposomes.

### 4.5. Antibacterial Activity

#### 4.5.1. Minimum Inhibitory Concentration (MIC) and Minimum Bactericidal Concentration (MBC)

Investigation of the activity of vancomycin-loaded nanoliposomes on vancomycin antimicrobial activity was held against bacterial populations with variable resistance mechanisms, multiple-drug resistance, and biofilm formation in comparison to vancomycin solution and blank liposomes.

The empty liposomes did not show any antibacterial activity against any of the tested bacterial strains. The vancomycin-loaded nanoliposomes showed remarkable minimization of the vancomycin MIC and MBC thresholds for all tested strains (unpaired *t*-test, *p* < 0.05) (Table 2).

The antimicrobial activity of the selected formula had the same potency for all implicated bacterial strains (MIC and MBC = 0.338 μg/mL) except for *S. epidermidis* ATCC 12228, which showed a slight reduction in sensitivity (MIC and MBC = 0.451 μg/mL).

Of particular interest, vancomycin-loaded nanoliposomes exhibited the same inhibitory and bactericidal effects on vancomycin-sensitive *S. aureus* (MRSA USA300) and the clinical isolates of heterogeneous vancomycin-intermediate-resistant *S. aureus* (hVISA). Meanwhile, the vancomycin solution showed a slight elevation in the MIC and a marked increase in the MBC for hVISA, which suggests that the resistant subpopulation of hVISA was mostly affected by vancomycin-loaded nanoliposomes with the same degree as that of the vancomycin-sensitive population.

#### 4.5.2. In Vitro Resistance Study

The abilities of different vancomycin-susceptible Staphylococcus strains (methicillin-resistant *Staphylococcus aureus* (MRSA USA300) and *Staphylococcus epidermidis* (ATCC 35984) to develop resistance against vancomycin upon repeated exposure to subinhibitory concentrations of optimum vancomycin-loaded nanoliposomes were examined. Changes in the MIC and MBC over the 25th passage were recorded in comparison to those for the vancomycin solution. Bacterial strains that had undergone multiple exposures to subinhibitory concentrations of vancomycin solution showed consistent elevation in their MICs and MBCs. By the end of the 25th passage, MRSA (USA300) and *S. epidermidis* (ATCC 35984) became vancomycin-resistant strains (VRSA, VRSE) with MICs and MBCs of 8.33 ± 3.61 μg/mL (Table 3).

**Figure 13:**
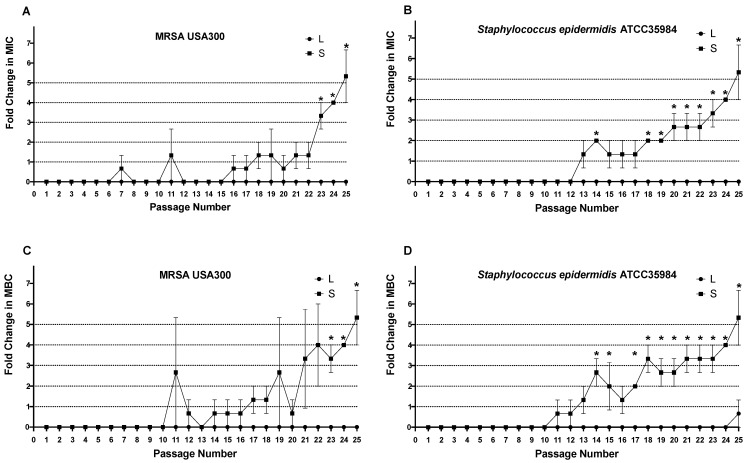
Serial-passages resistance experiment. Bacterial strains were serially passaged with concentrations of vancomycin liposomes (L) or vancomycin solution (S) over a period of 25 days. The broth microdilution method was used to determine the minimum inhibitory concentration (MIC) and minimum bactericidal concentration (MBC) of each passage. (**A**,**C**) MRSA USA300 fold change in MIC and MBC, respectively. (**B**,**D**) S.epidermidis ATCC35984 fold change in MIC and MBC, respectively. Data is presented as fold changes in MIC/MBC, and the error bars represent the standard error. * Indicates that the difference is significant at *p* < 0.05 (two-way ANOVA, Sidak’s post-test).

On the contrary, bacterial strains that were repeatedly exposed to subinhibitory concentrations of the optimized formula of vancomycin-loaded nanoliposomes exhibited constant vancomycin susceptibility without any changes in the MIC or MBC through all 25 passages, regardless of the statistically negligible changes in *S. epidermidis’* (ATCC 35984) MBC at the last passage (two-way ANOVA, Sidak’s post-test, *p* > 0.05) (Figure 13).

These findings highlight the significant controlling effect of antibiotic-loaded liposomes on evolution of selective bacterial resistance mechanisms against antibiotics.

#### 4.5.3. Effect of the Optimized Formula (Liposomes) on the Antibiofilm Activity of Vancomycin

**Figure 14:**
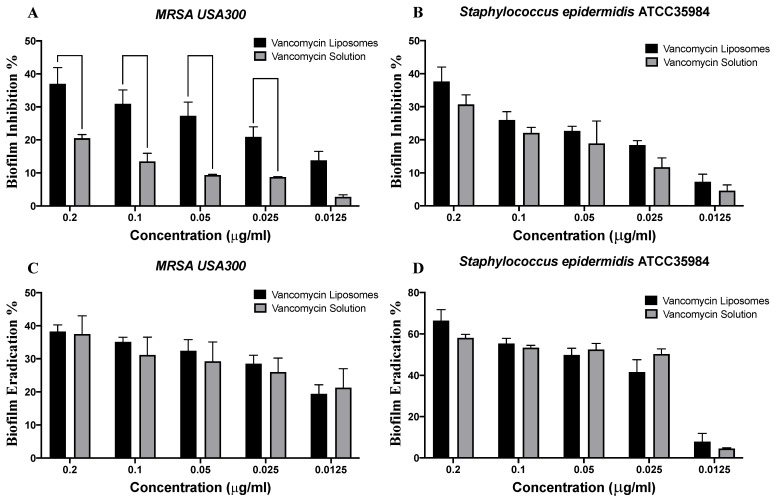
Antibiofilm activity. Effects of different sub-minimum inhibitory concentrations (MICs) (0.2–0.0125 μg/mL) of vancomycin liposomes and solution on methicillin-resistant Staphylococcus aureus (MRSA USA300) and Staphylococcus epidermidis ATCC 35984 biofilm formation. Results are expressed as mean biofilm inhibition percentages for MRSA, and Staphylococcus epidermidis ATCC (**A**,**B**) and mean biofilm eradication percentages for MRSA, and Staphylococcus epidermidis ATCC (**C**,**D**). Error bars represent the standard error.

The ability of the microbial pathogen to form biofilm plays a crucial role in augmenting virulence and resistance to various antimicrobial agents [57,86]. The antibiofilm activity of vancomycin-loaded nanoliposomes was compared to that of the vancomycin solution at concentrations below the determined MIC (0.2–0.0125 μg/mL). The vancomycin-loaded nanoliposomes recorded significantly higher biofilm inhibition activity than that of the vancomycin solution against MRSA USA300 (two-way ANOVA, Sidak’s post-test, *p* < 0.05) (Figure 14A). However, there was no significant difference in the inhibition activity against *Staphylococcus epidermidis* ATCC 35984 biofilm formation at any of the tested concentrations (two-way ANOVA, Sidak’s post-test, *p* < 0.05) (Figure 14B). Moreover, there was no significant difference between the vancomycin-loaded nanoliposomes and the vancomycin solution in biofilm eradication activity against the MRSA USA300 or *Staphylococcus epidermidis* ATCC 35984’s previously established biofilm at any of the tested concentrations (two-way ANOVA, Sidak’s post-test, *p* > 0.05) (Figure 14C,D).

### 4.6. In Vitro Eye Irritation Test

The HET-CAM was performed to detect potential irritation that may occur to ocular tissue upon application of vancomycin-loaded nanoliposomes against vancomycin solution, predicting probable safety.

The HET-CAM was utilized as a parallel model for the conjunctival tissue of the eye because the perfect vascularization of CAM tissue (arteries, veins, and capillaries) clearly demonstrates an inflammatory reaction in response to injury.

**Figure 15:**
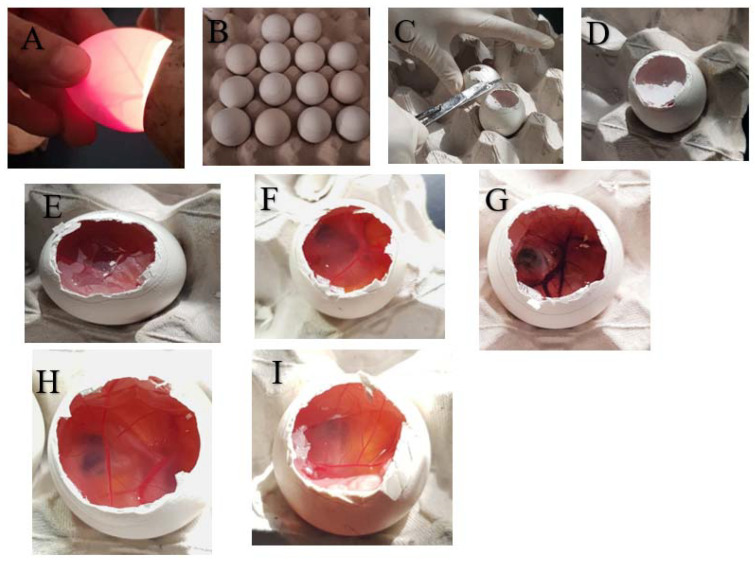
The hen’s egg test, chorioallantoic membrane (HET-CAM). Preparation steps (**A**–**E**): the eggs were candled to check their viability (**A**), air space was marked with a pencil (**B**), the eggshell around the air space was removed and CAM exposed (**C**,**D**), CAM was humified with saline (**E**). The observed responses on CAM upon application of different compounds (**F**–**I**): 0.9% NaCl (**F**), 10% NaOH (**G**), vancomycin solution (**H**), and vancomycin-loaded nanoliposomes (**I**).

Blood clotting and coagulation were present in the positive control (10% NaOH), as shown in Figure 15G. Nevertheless, 0.9% NaCl, the negative control, did not result in any irritating responses, as seen in the normal tissue vascularization (Figure 15F).

The vancomycin-loaded nanoliposomes and vancomycin solution produced the same outcomes as 0.9% NaCl (Figure 15H,I respectively), with neither exhibiting irritating effects on the CAM or any vascular injury. These results support the safety and nonirritating properties of vancomycin-loaded nanoliposomes as a potential platform to deliver vancomycin efficiently to ocular tissue.

## 5. Conclusions

In the present study, vancomycin was successfully loaded into liposomes using the active-loading method. A statistical design was used to determine the optimal vancomycin-loaded nanoliposomes with a high EE% and a small particle size. For the vancomycin-loaded nanoliposomes, significantly higher antibacterial activity and biofilm inhibition activity were recorded than those of vancomycin solution. Additionally, vancomycin-loaded nanoliposomes effectively prevent development of MRSA selective vancomycin resistance. These findings highlighted the significance of efficient loading and successful intracellular delivery of vancomycin in breaking development of resistance mechanisms, which is fundamental to saving its antimicrobial activity and therapeutic use. Further studies for elucidating the key formulation factors required for preparation of ocular nanoliposomes for sustained delivery of vancomycin into the eye tissue are presently being investigated.

## Figures and Tables

**Table 1:** Results of vancomycin entrapment efficiency (EE%), particle size, zeta potential, and polydispersity index (PDI).

Formulations	Cholesterol %	Drug:Lipid Ratio	Number Drug Solution Exchange	Entrapment Efficiency % ± SD	Particle Size (nm) ± SD	Zeta Potential (mV) ± SD	PDI
F1	10	0.2	3	32.14 ± 2.0	141 ± 2.7	−21 ± 1.8	0.20
F2	30	0.05	3	37.06 ± 1.5	282 ± 8.0	−38 ± 0.7	0.37
F3	10	0.05	1	50.34 ± 2.4	149 ± 9.2	−30 ± 1.0	0.21
F4	10	0.05	3	33.58 ± 1.7	153 ± 3.2	−27 ± 0.9	0.18
F5	10	0.2	1	41.09 ± 2.5	157 ± 2.0	−28.5 ± 1.2	0.18
F6	30	0.2	3	30.56 ± 1.7	353 ± 3.5	−41 ± 0.5	0.42
F7	30	0.2	1	32.09 ± 1.5	316 ± 10	−32 ± 2.0	0.30
F8	30	0.2	2	41.24 ± 8.5	307 ± 5.3	−18.5 ± 1.2	0.23
F9	10	0.05	2	53.46 ± 2.9	148 ± 4.0	−30 ± 0.7	0.18
F10	20	0.05	2	65.00 ± 4.0	155 ± 5.4	−34 ± 1.5	0.25

**Table 2:** Minimum inhibitory concentrations (MICs) and minimum bactericidal concentrations (MBCs) against tested bacterial strains.

Bacteria	Description	Vancomycin Solution		Vancomycin-Loaded Liposomes	
		MIC (μg/mL)	MBC (μg/mL)	MIC (μg/mL)	MBC (μg/mL)
*Staphylococcus aureus* (MRSA USA300)	Methicillin-resistant/Vancomycin senstive	1.563 ± 0.0	2.083 ± 0.9	0.338 ± 0.0	0.338 ± 0.0
*Staphylococcus aureus* (hVISA OP933674)	Heteroresistant vancomycin-intermediate Staphylococcus aureus	2.083 ± 0.9	6.25 ± 0.0	0.338 ± 0.0	0.338 ± 0.0
*Staphylococcus epidermidis* ATCC 35984	Biofilm positive	1.563 ± 0.0	1.563 ± 0.0	0.338 ± 0.0	0.338 ± 0.0
*Staphylococcus epidermidis* ATCC 12228	Biofilm negative	1.563 ± 0.0	1.563 ± 0.0	0.451 ± 0.2	0.451 ± 0.2

**Table 3:** Vancomycin susceptibilities of different Staphylococcus strains in terms of changes in minimum inhibitory concentration (MIC) and minimum bactericidal concentration (MBC) with several exposures to subinhibitory concentrations of vancomycin-loaded nanoliposomes and vancomycin solution; table illustrates MIC and MBC values of first and last passages.

Bacteria	Number of Passages	Vancomycin Solution				Vancomyci-Loaded Liposomes			
		Start Passage		Last Passage		Start Passage		Last Passage	
		MIC (μg/mL)	MBC (μg/mL)	MIC (μg/mL)	MBC (μg/mL)	MIC (μg/mL)	MBC (μg/mL)	MIC (μg/mL)	MBC (μg/mL)
*S. aureus* (MRSA USA300)	25	1.563 ± 0	2.083 ± 0.9	8.33 ± 3.61	8.33 ± 3.61	0.338 ± 0	0.338 ± 0	0.338 ± 0	0.338 ± 0.0
*S. epidermidis* ATCC 35984	25	1.563 ± 0	1.563 ± 0.0	8.33 ± 3.61	8.33 ± 3.61	0.338 ± 0	0.338 ± 0	0.338 ± 0	0.451 ± 0.195

## Data Availability

Not applicable.

## Supplementary Materials

The following supporting information can be downloaded at: https://www.mdpi.com/article/10.3390/pharmaceutics15061636/s1, Figure S1: (A) Slide A, G2 dialysis cassettes, (B) Cassette cracking, (C) Entire cassette components; Figure S2: Ultrafiltration membrane; Figure S3: (A–C) modeling and applying of the PCR sealing film to the inner side of the plastic frame using a paperclip to prepare the inner liposomal champers; Figure S4: (A,B) represent the two halves of the cassette, each one composed of a plastic frame and silicon gasket, where the ultrafiltration membrane was aligned in between. (C) the chamber where the ultrafiltration membrane glossy was inside assigned as a drug chamber. (D) the other cassette chamber was assigned as liposomes chamber; Figure S5: (A) cassette was assymbled and compressed by clamps. (B,C) top and side view of cassette upon epoxy hardening.
