# Supplementary material for: Controlling the Evolution of Selective Vancomycin Resistance through Successful Ophthalmic Eye-Drop Preparation of Vancomycin-Loaded Nanoliposomes Using the Active-Loading Method
Source: Pharmaceutics. 2023 May 31;15(6):1636. doi: 10.3390/pharmaceutics15061636 (PMC10303023; doi:10.3390/pharmaceutics15061636)
Supplement: Supplementary file 1 [file pharmaceutics-15-01636-s001.zip › pharmaceutics-2334890-supplementary.pdf]

Content:

- Liposomal cassette construction.
- Cassette cleaning and storage.
- Reagent Setup.

Liposome dialyzer cassette construction

- Two G2 dialysis cassettes were cracked from their side using a metallic card (Figure S1 A, B). Each cassette is composed of two halves of the plastic frame, one silicon gasket, one plugin, and two dialysis membranes (Figure S1 C).

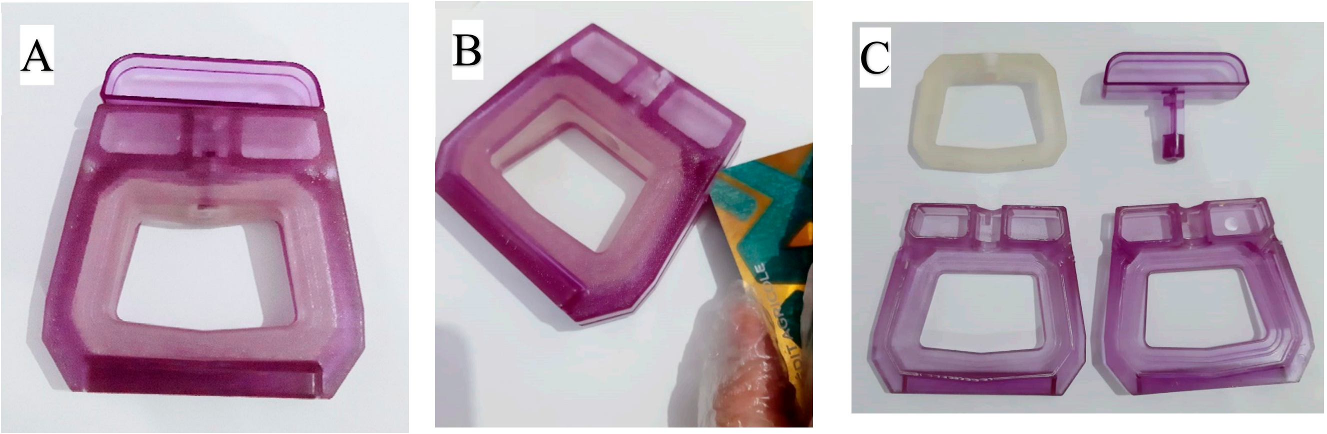

Figure S1) A) Slide A, G2 dialysis cassettes, B) Cassette cracking, C) Entire cassette components.

Only two halves of the plastic frames, the two silicon gaskets of the two cassettes, the two plugins of the two cassettes, and an ultrafiltration membrane (NMWL 100.000 KDa, Millipore, USA) were used for constructing the liposome dialysis cassette.

- Using the original cassette dialysis membrane, the ultrafiltration membrane was modeled to adapt cassette shape and size (Figure S2). Afterward, it was washed with plenty amount of Milli Q water with the glossy surface facing the water. As guided by the manufacturer, water was changed thrice at one hour for glycerin removal from the membrane.

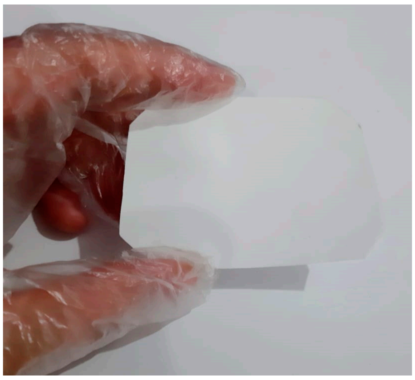

Figure S2) Ultrafiltration membrane

- For constructing cassette chambers, a *polymerase chain reaction* (PCR) sealing film (Applied Biosystems, USA) was modeled and applied to the inside of the plastic frame to seal the original dialysis window, where the sealing film was pushed inside the plastic frame grooves using a paper clip to affirm its attachment (Figure S3 A, B). PCR sealing film was used where the sealing film's non-sticky side would be the inner side of the constructing chambers, and its sticky side would be the external surface of these chambers. Also, PCR sealing film was applied to the outer side of the plastic frame to protect the outward sticky surface of the chamber wall from dust or solid matter, maintaining its window clarity (Figure S3 C).

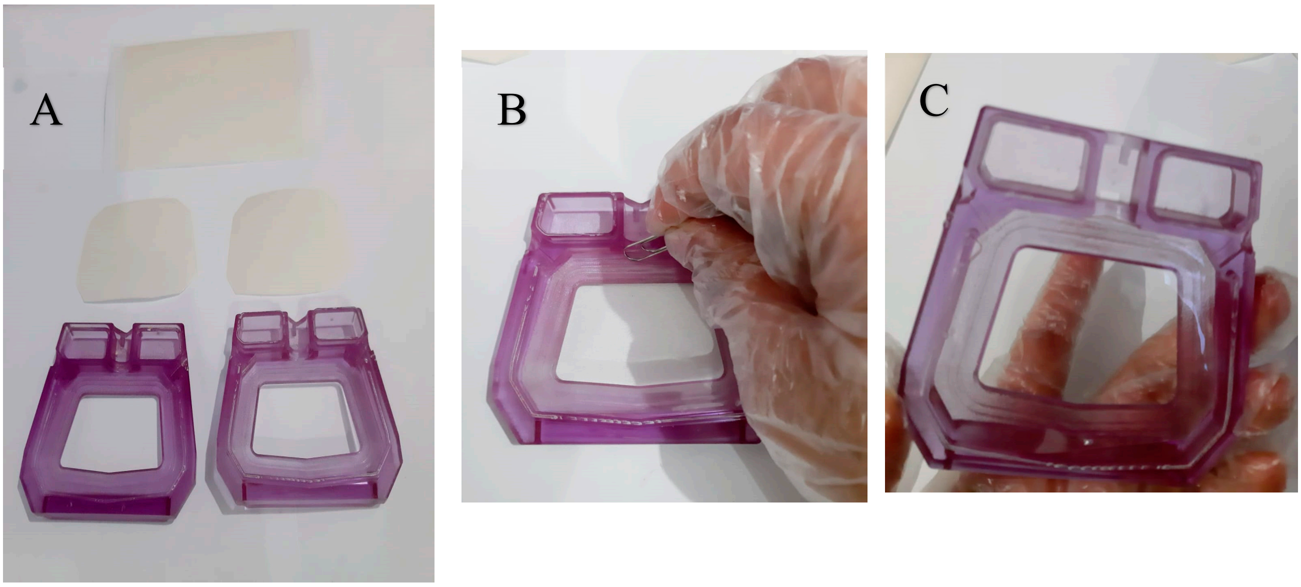

Figure S3) A, B, and C modeling and applying of the PCR sealing film to the inner side of the plastic frame using a paperclip to prepare the inner liposomal champers.

- Each silicon gasket was aligned to one part of the plastic frame, and the two parts were assembled while an ultrafiltration membrane was placed in between, as illustrated in (Figure S4). During cassette assembly, the chamber that would contain the membrane's glossy surface inside was assigned as the drug chamber, and the other one as the liposomal chamber.

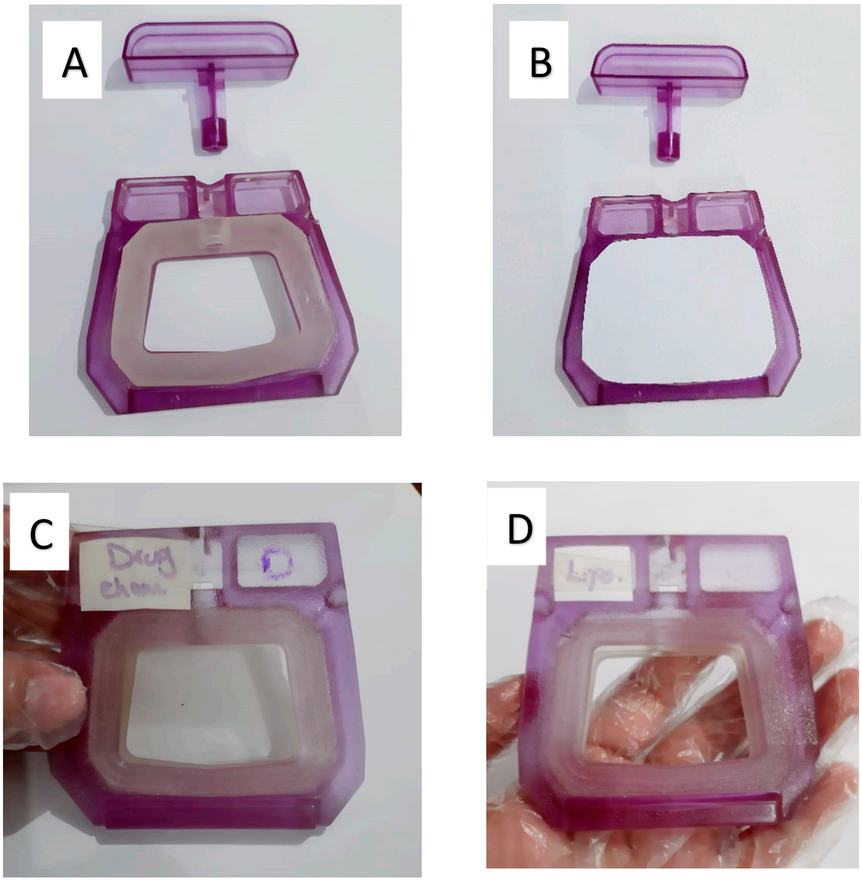

Figure S4) A and B represent the two halves of the cassette, each one composed of a plastic frame and silicon gasket, where the ultrafiltration membrane was aligned in between. C the chamber where the ultrafiltration membrane glossy was inside assigned as a drug chamber. D the other cassette chamber was assigned as liposomes chamber.

- . Two burette clamps were used to fix and compress the cassette on both sides to be ready for epoxy application (Figure S5, A). Waterproof Epoxy (Fevicol, India) was prepared according to the manufacturer's technique and applied using a plastic knife to cover all cassette sides (Figure S5, B&C). The thickness of the plugin's upper end was decreased using a heated metal palette knife to ease its insertion and removal during the loading process.

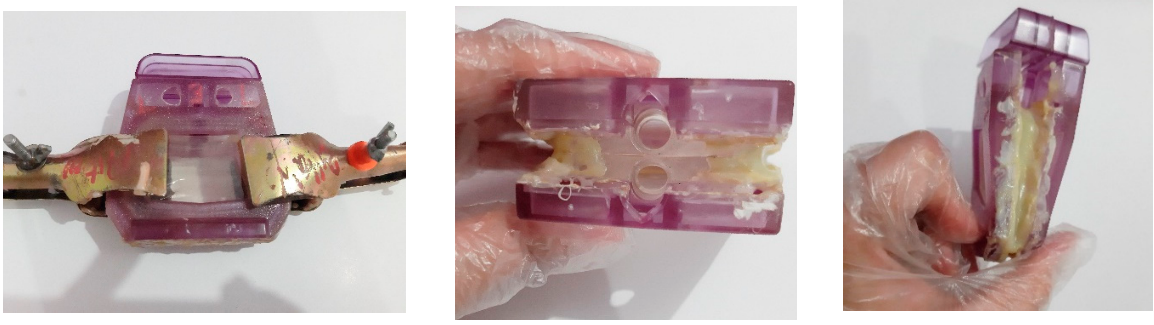

(A) (B) (C)

Figure S5) A cassette was assymbled and compressed by clamps. B and C top and side view of cassette upon epoxy hardening.

Cassette cleaning and storage

To clean the cassette, a heated solution up to 55°C of 1% tween 20 was used to wash the cassette inside and the ultrafiltration membrane from lipids with tumbling at 80 rpm for 10 minutes, followed by water and 0.1% sodium hydroxide solution. Then, the cassette chambers were washed thoroughly with water, afterward by the buffer solution. Ultrafiltration membrane should be allowed to be equilibrated with the buffer solution for at least 5 minutes.

For storage, cassette chambers were filled after cleaning with 20% (V/V) ethanol and refrigerated to preserve and prevent dryness of the membrane.

Reagent Setup:

- **Lipid stock solutions preparation**

  100mM stock solutions of cholesterol and soy lecithin were prepared for the following molar ratio (1:9), (2:8), and (3:7), respectively.

  The amount of soy lecithin and cholesterol was calculated using the following formula:

  (Weight)g = Molecular weight * Molar fraction * Molarity(M) * volume(L)

  Lipids were desiccated before use and accurately weighed, then cholesterol and soy lecithin, each dissolved in half of the final intended volume using

chloroform. Solutions were divided into small aliquots for single use and stored at -80 C.
For liposome preparation, equi-volumes of soy lecithin and cholesterol are mixed just before use.

- **Tris buffer preparation**

  Tris buffer (200mM, pH 9 at 25°C) was prepared by mixing pre-prepared Tris base and Tris-HCl solutions according to Sigma buffer reference center tabulated ratio at our selected pH and temperature.
  Both tris base and tris-HCl were desiccated before use and accurately weighed—buffer solution was used within one week from its preparation.

- **Sephadex preparation:**

  Sephadex G 50 resign gel was prepared by allowing sephadex powder to swell at an excess amount of 350mM NaCl solution at 90 °C for 1 hour, considering the bed volume of sephadex G 50 fine is 9-11 ml/g.
  After swelling, air bubbles were removed via sonication pulse of the bath sonicator.

  For cleaning of sephadex, sephadex was washed overnight with a heated solution (90 °C) of 1M acetic acid and 1% tween 20 followed by one column of water, 20% ethanol, 0.1 NaOH, then sephadex was equilibrated with at least five columns of the eluting solution (350 mM NaCl) with UV spectrophotometer monitoring to ensure a stable baseline.

  Sephadex is stored in 20% (V/V) ethanol and refrigerated to avoid microbial growth.
